# The impact of the COVID-19 virus and pandemic on healthcare utilization, access, delivery, experiences, and outcomes in the spinal cord injuries/dysfunction population: A scoping review study

**DOI:** 10.1371/journal.pone.0297384

**Published:** 2024-02-22

**Authors:** Arrani Senthinathan, Stephanie Cimino, Susan B. Jaglal, B. Catharine Craven, Karen Tu, Sara Guilcher

**Affiliations:** 1 Leslie Dan Faculty of Pharmacy, University of Toronto, Toronto, Ontario, Canada; 2 Rehabilitation Science Institute, Temerty Faculty of Medicine, University of Toronto, Toronto, Ontario, Canada; 3 Institute of Health Policy Management and Evaluation, University of Toronto, Toronto, Ontario, Canada; 4 Department of Physical Therapy, Temerty Faculty of Medicine, University of Toronto, Toronto, Ontario, Canada; 5 KITE (Knowledge Innovation Talent Everywhere), Toronto Rehabilitation Institute - University Health Network, Toronto, Ontario, Canada; 6 Department of Medicine, Faculty of Medicine, University of Toronto, Toronto, Canada; 7 Spinal Cord Rehabilitation Program, Toronto Rehabilitation Institute – University Health Network, Toronto, Canada; 8 Department of Family and Community Medicine, University of Toronto, Toronto, Ontario, Canada; 9 North York General Hospital, Toronto, Ontario, Canada; 10 Toronto Western Family Health Team, University Health Network, Toronto, Ontario, Canada; Calcutta National Medical College, Government of West Bengal, INDIA

## Abstract

**Background:**

Individuals with spinal cord injuries or disease (SCI/D) require frequent healthcare services. The COVID-19 pandemic may have impacted healthcare. Furthermore, due to secondary health conditions and comorbidities persons with SCI/D are at increased risk of experiencing severe symptoms or outcomes if infected with the COVID-19 virus. It is unclear to what extent research has investigated the pandemic and virus impacts on the SCI/D population.

**Objective:**

To identify and summarize what is reported in the literature on the impact the COVID-19 virus and pandemic had on healthcare, health outcomes, and experiences in the adult SCI/D population.

**Methods:**

Electronic databases and grey literature were searched for articles that included an adult population with a SCI/D and investigated the impact the COVID-19 virus and pandemic had on healthcare-related outcomes and experiences. Articles were double screened, and data were extracted, and synthesized to provide a descriptive summary of the findings.

**Results:**

Twenty-four studies were included in this review with eight qualitative, fifteen quantitative, and one mixed methods study. Sixteen studies investigated healthcare utilization/access; nine investigated care delivery, nine investigated patient outcomes, and eight investigated patient experiences, with multiple studies spanning different categories of investigation. The pandemic was detrimental to healthcare utilization, access, and outcomes, but no studies quantified these changes. Virtual care was well-received by the SCI/D population to maintain continuity of care. The SCI/D population had issues with maintaining caregiving support. It was unclear if the COVID-19 virus infection impacted individuals with SCI/D differently than the general population.

**Conclusions:**

This scoping review found the pandemic negatively impacted multiple aspects of healthcare in individuals with SCI/D, however further investigation on health outcomes is required. More research, particularly large-scale quantitative studies, investigating healthcare access, utilization, and delivery, as well as patient outcomes and experiences is needed to improve care in the SCI/D population post-pandemic onset.

## Introduction

The COVID-19 pandemic has led to unprecedented and significant shifts worldwide in healthcare delivery [1–3], services, utilization [4, 5], access [6], outcomes [7], and experiences [8–10]. Individuals who require frequent interactions with the healthcare system were likely impacted by these rapid changes. Specifically, individuals with spinal cord injuries or disease (SCI/D) were likely impacted, as they often rely heavily on healthcare services, mainly due to the presence of co-morbid secondary health condition and multimorbidity that require ongoing care management [11–13]. As such, individuals with SCI/D may be especially impacted by these shifts in healthcare due to the pandemic.

During the pandemic, many health systems transitioned towards virtual care services to ensure continuity of care and sustained access to health services [6]. Although, virtual care may be beneficial for those who experience physical challenges when accessing care, it may also exacerbate inequities for those with disabilities, who face barriers accessing and using technology [2, 6, 14]. Furthermore, SCI/D care often requires physical hands on assessments, which cannot be conducted virtually [2, 14]. It has also been suggested that individuals with SCI/D may experience the COVID-19 infection differently than the general population [15]. Due to an increase in comorbidities and secondary conditions, individuals with SCI/D may be at an increased risk for severe symptoms or increased negative health outcomes if they contract the COVID-19 virus [15]. As a preventative measure, individuals with SCI/D may choose to limit in-person visits, resulting in a decrease of in-person assessments that may be required for appropriate care [2, 16–18].

It is important to understand the impact the pandemic had on individuals with SCI/D to help guide future research, as well develop recommendations to improve care for individuals with SCI/D as the pandemic continues to progress, and new variants emerge. As such, we undertook a scoping review to identify literature that has described or investigated healthcare utilization, access, and delivery, as well as patient outcomes and experiences for individuals with SCI/D due to impact of the COVID-19 pandemic, and infection of COVID-19 virus. Specific objectives were: 1) to examine the extent and type of literature pertaining to how healthcare was affected by the pandemic and how COVID-19 virus infection impacted individuals with SCI/D; 2) to examine and describe changes in healthcare utilization and access, care delivery, patient outcomes, and patient experiences among individuals with SCI/D during the COVID-19 pandemic; 3) to identify any gaps in the literature in order to propose future studies and/or make recommendations regarding future health service needs/recommendations.

## Methods

This scoping review was conducted and designed based on the methodology developed by Joanna Briggs Institute Methodology for Scoping Reviews [19]. The Preferred Reporting Items for Systematic Reviews and Meta-Analyses extension for scoping review (PRISMA-ScR) checklist was used as the reporting guideline for this scoping review [20].

### Protocol and registration

The protocol for this scoping review was registered on November 11, 2022 on the Open Science Framework Registries (https://osf.io/xf8ru/).

### Eligibility criteria

This scoping review focuses on healthcare-related topics in the SCI/D population during the COVID-19 pandemic. SCI/D includes both traumatic spinal cord injury (TSCI) and non-traumatic spinal cord disease (NTSCD). TSCI are the result of catastrophic events involving direct or indirect external force to the spinal cord, such as falls or motor vehicle collisions [21]. NTSCD are not caused by an external force, but usually the result of tumours, infections, neurodegenerative diseases, or post-surgical complications that affect the spinal cord [22, 23]. NTSCD can be further divided into degenerative and non-degenerative causes [23–27].

#### Inclusion criteria

To be included in this scoping review, articles in any language were required to: (1) have an adult population (≥18 years) with SCI/D (TSCI and/or NTSCD); (2) include components and topics related to healthcare; and (3) focus on the COVID-19 virus and/or pandemic. To meet the first inclusion criteria, all participants had to be over the age of 18 with SCI/D or separate results for those over the age of 18 must be presented. Paediatric populations often exhibit different healthcare utilization, hence we chose to limit this initial search to the adult population. If non-SCI/D populations were included in the study, the article must also have presented separate results for the SCI/D population or at least 50% of the participants had to have SCI/D to ensure adequate resulting pertaining to the SCI/D population. Individuals with Multiple Sclerosis (MS), and Poliomyelitis (Polio) or spinal muscular atrophy, amyotrophic lateral sclerosis (ALS), ankylosing spondylitis, and spondylarthritis were excluded from the review as they have different healthcare needs and pathophysiology compared to other SCI/D diagnoses. To meet the second criteria, the articles did not have to have a healthcare-related topic as the main focus, however, they needed to discuss within the results healthcare related components, which included healthcare utilization or access, healthcare services/delivery, patient experiences/outcomes, and/or impact of COVID infection on individuals with SCI/D. Basic science research, including studies focused on the molecular or cellular pathophysiology of the COVID-19 virus in individuals with SCI/D, or research investigating SCI/D as an outcome/complication/symptom of COVID-19 virus/infection and/or vaccine were excluded, due to an absence of direct application to the healthcare system. Furthermore, epidemiology papers focused on rates of referrals or procedures of radiotherapy, neurosurgery and spinal surgery were also excluded. To meet the third criteria, the articles must have focused on the COVID-19 virus and/or pandemic.

#### Exclusion criteria

Articles were excluded if they met at least one of the following criteria: books, book chapters, opinion pieces, editorials, commentaries, case studies (less than 10 cases/persons), conference abstracts, narrative reviews, protocols, guidelines, or if we were unable to access the full-text. We also excluded conference abstracts and study protocols to ensure all included articles presented finalized results.

### Search methods

The searches were developed by an applied health sciences researcher (AS) with consultation among the team members (SJTG, SRC), as well as a librarian scientist. Four electronic databases were searched on November 24, 2022: MEDLINE (Ovid Interface), EMBASE (Ovid Interface), CINAHL Plus (EBSCOhost Interface), and Scopus (Elsevier). The Ovid MEDLINE search was reviewed by another experienced health science librarian using PRESS (Peer Review of Electronic Search Strategies).^24^ The searches were constructed using the concepts of “SCI/D” AND “healthcare” AND “COVID-19”. The search was translated into the databases using each platforms’ command language and controlled vocabulary, where applicable. No other limits were applied on the searches to ensure we identify potentially applicable studies. The full database search strategies, copied and pasted exactly as run, can be found in S1 Appendix. The electronic database searches were supplemented by grey literature searches conducted November 26, 2022, on relevant websites, including the World Health Organization, Spinal Cord Injury Research Evidence (SCIRE), Dissertations and Theses Global (ProQuest), Praxis Spinal Cord Institute, International Spinal Cord Society and MedRxiv.

### Selection process

The articles identified from the database searches were uploaded into Covidence^®^. Covidence^®^ was used to de-duplicate and screen articles to ensure no duplicate citations.^25^ Three reviewers (AS, SRC, SJTG) screened 50 titles and abstracts to ensure good agreement (>80% agreement) [28]. The reviewers had over 90% agreement. No revisions or clarifications to the eligibility criteria were required, and the remaining titles and abstracts were double screened.

**Fig 1:**
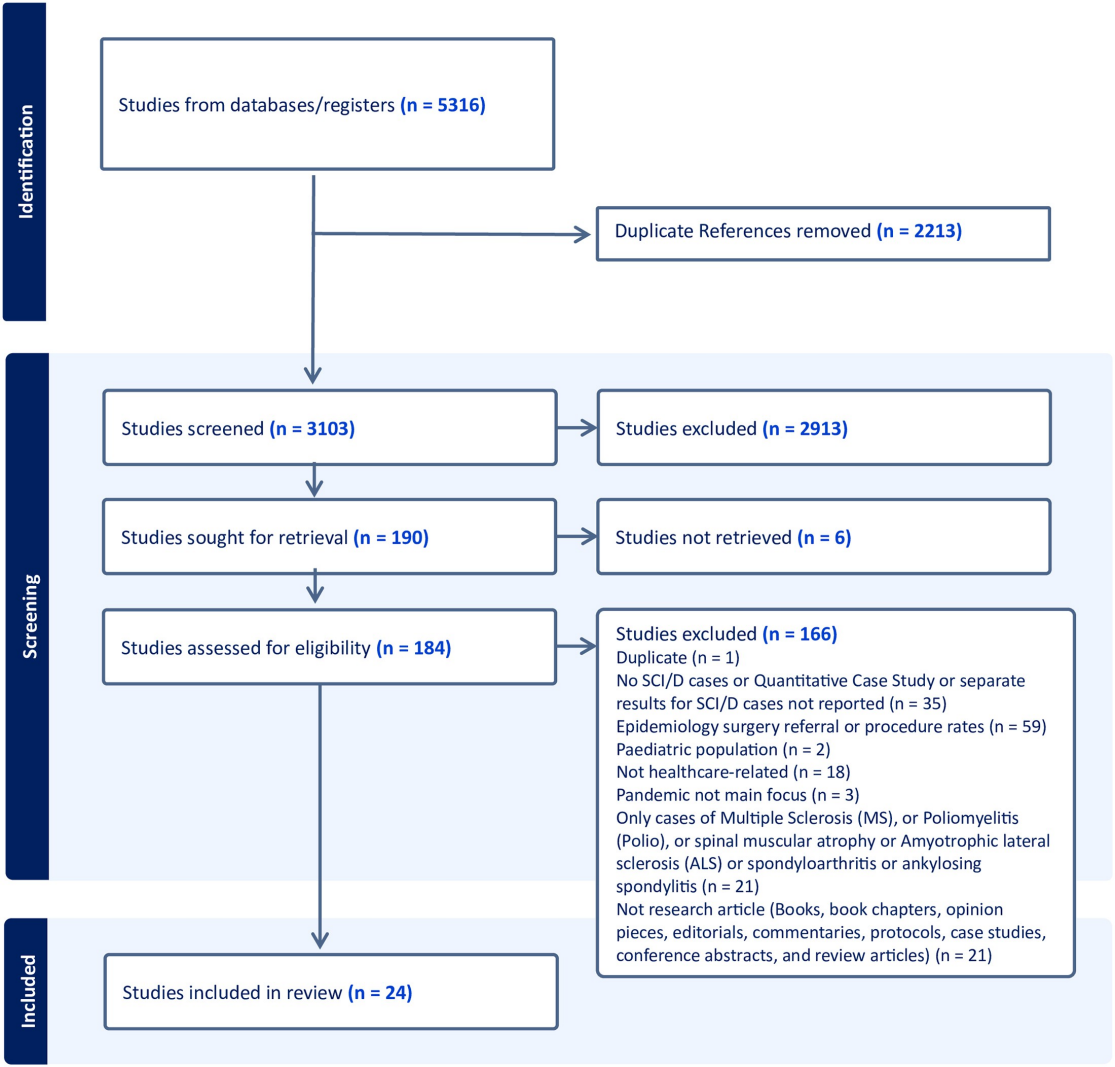
PRISMA flow diagram of articles included in review.

All disagreements were resolved through consensus. Following the title and abstract screen, three reviewers (AS, SRC, and SJTG) completed a test screen of ten full-text articles to ensure good agreement and that all criteria were being interpreted and applied in the same way. The reviewers had 80%agreement and the remaining full-text articles were double screened. All disagreements were resolved through consensus. The PRISMA flow diagram documenting the records identified, included, and excluded can be found in Fig 1.

### Data charting process

A data extraction table was created in Covidence^®^ to facilitate the extraction process. Three team members conducted the double extraction. All articles were double extracted by two team members independently. Any disagreements in the extraction process were resolved through consensus.

### Data items

The extracted variables included: general article information (title, authors, year of publication, journal, funding), study characteristics (objective, study design, data source, eligibility criteria, outcomes, country, setting), and population characteristics (sample size, age, sex, gender, ethnicity/race, income, education, marital status, household composition, employment status, comorbidities, injury characteristics, type of SCI/D). Additionally, healthcare and health-related study outcomes and findings (results and key findings, conclusions) were extracted.

### Synthesis methods

Data were synthesized descriptively, including the study designs, data source, countries, years of publication, population characteristics, and study outcomes/findings. Articles were categorized by one team member (AS) for healthcare topic.

## Results

### Study selection

A total of 5,316 articles were identified from the database searches, with 2,213 duplicates removed, and 3,103 remaining following de-duplication. At the title and abstract level of screening, 2,913 articles were excluded, with 186 remaining for full-text screening. Of the 186 full-text articles screened, 162 were excluded. A final total of 24 studies were included in the scoping review (see Fig 1 for PRISMA Diagram).

### Study country and setting

Study country and setting are listed in Table 1. Nine studies were conducted in North American countries [2, 9, 29–35], five studies were conducted in European countries [17, 36–40], three in Asian countries [41–44], one in Australia [45], and one in South America [46]. Four studies were conducted internationally spanning multiple countries and continents [47–50]. Eighteen studies were conducted in outpatient settings in the community [2, 9, 17, 29–35, 39–41, 43, 45, 46, 48, 49]. three were conducted in in-patient settings [36, 38, 44], two were conducted in both outpatient and in-patient settings [47, 50], and one study’s setting was not described [42]. Fourteen studies were published in 2022 [9, 17, 31–35, 39–41, 43–46], seven were published in 2021 [2, 30, 36, 37, 42, 47–49] and three were published in 2020 [29, 38, 50].

**Table 1:** Summary of scoping review findings.

Author (year), setting, country	Objective	Study Design (data source)	Healthcare focus (Y/N), Category	Key Findings and Conclusions Related to Healthcare
Bhuva et al. (2020), Out-patient, United States	To investigate the implementation and satisfaction of telemedicine in a physical medicine and rehabilitation spine practice during COVID-19.	Quantitative Prospective Cohort (survey)	Y, Care Delivery	97.6% patients satisfied with their virtual appointment 87% patients reported no issues during their telemedicine encounter, 8% had video issues and 3% had audio issues 64.5% patients preferred virtual over in-person appointments
D’Andrea et al. (2020), In-patient, Italy	To evaluate whether clinical course of COVID-19 can be more severe in people with spinal cord injury than in able-bodied individuals.	Quantitative Observational case—control (clinical data)	Y, Patient Outcomes	Compared to control group, patients with spinal cord injury were older and had a higher prevalence of comorbidities, yet no significant differences in COVID-19 clinical features and treatment were found Both groups treated in non-intensive care with no deaths
Stillman et al. (2020), In-patient & Out-patient, International	To query the international spinal cord medicine community’s engagement with and response to the COVID-19 pandemic	Quantitative Observational (survey)	Y, Patient Outcomes	Differences in COVID-19 screening for SCI/D population varied substantial across countries especially based on availability of screen kits
Barrows & Goldstein (2021), Out-patient, United States	To assess virtual care for Veterans with spinal cord injuries and disorders (SCI/D) in Veterans Affair centers.	Quantitative Retrospective Descriptive (health database)	Y, Care Delivery	Virtual care was a useful tool to support SCI/D care, as there was unprecedented increase and use during the pandemic However not all SCI/D care can be performed virtually
Battisti et al. (2021), In-patient, Italy	To evaluate vertebral fractures and their effect on survival in COVID-19 and non-COVID-19 patients.	Quantitative Retrospective Cohort (chart review)	Y, Patient Outcomes	Mortality risk associated with vertebral fractures only in non-COVID group, and not in COVID group, after adjustment for age, sex, and bone density
Crawford et al. (2021), Out-patient, United States	To assess the relevance of telemedicine to interventional spine procedure planning.	Quantitative Retrospective (chart review)	Y, Care Delivery	Pre-procedural treatment plans established by telemedicine did not change for 87% of individuals after in-person assessments Interventional procedure plans developed during telemedicine visits are estimated to be accurate in 79–94% of cases
Gustafson et al. (2021), In-patient & Out-patient, International	To follow-up with and re-query the international spinal cord community’s response to the Coronavirus Disease 2019 (COVID-19) pandemic by revisiting questions posed in a previous survey and investigating new lines of inquiry.	Mixed-Method (survey)	Y, Healthcare access & utilization; Patient outcomes; Care delivery; Patient experiences	25.6% of facilities were not accepting patients with positive COVID-19 diagnosis 67.5% reported pandemic resulted in fewer spinal cord injury admissions 56.9% found it difficult to provide usual level of care for spinal cord injury Decreases in therapy and caregivers were reported, as well as unexpected patient complications due to COVID-related barriers Over 60% reported need for more education on how COVID impacts people with disabilities, and patient rehabilitation 81.2% intentionally include more virtual visits 63.2% reported with telemedicine some needs are met, but protocols for privacy/safety/best practice are need
Hatefi et al. (2021), unknown, Iran	To determine the challenges of spinal cord injury patients during the COVID19 pandemic.	Qualitative (interview)	N, Healthcare access & utilization; Patient experiences	Since the onset of the COVID-19 outbreak, spinal cord injury patients expressed challenges with access to services, and how to get informed about the infection.
Mikolajczyk et al. (2021), Out-patient, International	To understand how resilience, access to personal care attendants and medical supplies, and concerns about medical rationing, finances, and social isolation are related to overall and mental health in individuals with spinal cord injury in the context of the COVID-19 pandemic.	Quantitative Observational Cross-sectional (survey)	N, Healthcare access & utilization	Belief and/or concerns of medical rationing impacted participants overall health, mental health, anxiety, depressive symptoms, and quality of life.
Monden et al. (2021), Out-patient, International	To describe impact of the pandemic on the SCI community focused on medical discrimination and medical rationing, access to personal care attendants and medical supplies, and overall and mental health	Quantitative Observational Cross-sectional (survey)	N, Healthcare access & utilization	60% of participants reported pandemic had negative impact on care they received at home 59% of participants reported the pandemic negatively impacted their access to medical supplies. 68% reported the pandemic has had a negative impact on their overall health
Bhattarai et al. (2022), Out-patient, Nepal	To explore the impacts of COVID-19 on multiple aspects of the lives of individuals living with spinal cord injury in Nepal.	Qualitative (focus groups)	N, Patient outcomes; Healthcare access & utilization; Patient Experiences	Inability to receive healthcare services and rehabilitation during lockdown resulted in or worsened secondary conditions Participants reported inadequate resources/supplies, unavailable caregivers, and physical barriers to care during pandemic Rural areas had difficulty accessing medications and health care supplies needed for their symptom management and self-care during pandemic
Dean et al. (2022), Out-patient, Canada	To explore individuals with spinal cord injury experiences with and perceptions towards virtual services during the COVID-19 global pandemic in British Columbia, Canada.	Qualitative (survey)	Y, Care Delivery	Virtual care was convenient, accessible, and effective, saved time and money, and alleviated anxieties of contracting COVID-19 Virtual care was less personal with difficulties building rapport, reading body language and feeling rushed Technology issues included access, digital literacy, privacy, technology cost, internet connectivity, and age of devices. In-person care was still needed for some services
Faleiros et al. (2022), Out-patient, Brazil	To understand the perceptions of people with spinal cord injury in Brazil post-pandemic.	Qualitative (survey)	N, Healthcare access & utilization	Participants reported a lack of access to treatments and rehabilitation
Hearn et al. (2022), Out-patient, United Kingdom	To explore the impact of the COVID-19 pandemic on individuals living with spinal cord injury using a qualitative inquiry approach, including changes to daily life, personal challenges, positive experiences, and potential future impact of the pandemic.	Qualitative (survey)	N, Healthcare access & utilization; Patient outcomes; Care delivery	Individuals reported lack of access to health services and specific supports, as well as cancelled consultations Concerns regarding physical health, including deterioration in physical strength and increase in pain were reported Access to personal protective equipment and spinal cord injury-related equipment, such as wheelchairs, was impeded, which had implications for self-management activities such as bowel care and pain management, which could lead to complications. Increased utilization of online/ phone consultations with medical professionals was beneficial and improved healthcare access
Hill et al (2022), Out-patient, United States	To understand how COVID-19 has affected the daily lives of people living with cervical spinal cord injury.	Qualitative (interview)	N, Healthcare access & utilization; Patient outcomes; Care delivery; Patient experiences	60% described temporary and long-term changes to caregiving Concern that caregivers with multiple clients increase exposure risk Many participants continued to use caregivers outside of family members, others stopped using external caregiving services at least temporarily, which increased demand and stress for families Many experienced restricted access to healthcare services and supplies Telemedicine promoted feelings of safety during the pandemic, reduced some barriers to healthcare, and was recommended for basic healthcare needs, however not all care conducive to telemedicine Telemedicine supported continued access to care, though there were some concerns with digital literacy
Kaner et al. (2022) Out-patient, United States	To identify patient characteristics associated with a positive COVID-19 test. The secondary outcome was to identify patient characteristics associated with mortality from COVID-19	Quantitative Retrospective Cohort (health database)	Y, Patient Outcomes	African Americans and increased Body Mass Index had increased odds of testing positive for COVID-19 Increased age and smokers had increased odds of mortality with positive COVID-19 test (1.046 (1.003–1.090)) No association was found between neurologic level of injury and positive COVID-19 test or increased mortality Fatality rate for persons with SCI and a positive test for COVID-19 was 12%
Lakhani et al. (2022), Out-patient, Australia	To investigate the impact of lifting social distancing restrictions on priority domains for people with spinal cord injury residing in the state of Victoria, Australia.	Quantitative Longitudinal (survey)	N, Healthcare access & utilization; Patient outcomes	Participants had a significant increase (p<0.05) in secondary health conditions, as the pandemic progressed based on data collected September and October 2020 compared to April 2021 and May 2021 Participants indicating health services were inaccessible during the first 6-months of the pandemic and end of 2020/early 2021. Fewer participants reported inaccessible services in April 2021 and May 2021 compared to September and October 2020 Increases in health issues may be a result of poor access to health services during the period of lockdown
Matsuoka & Sumida (2022), Out-patient, Japan	To clarify the effect of the COVID-19 pandemic on health-related quality of life (HRQOL) in home-based patients with spinal cord injury.	Quantitative Descriptive (survey)	N, Healthcare access & utilization; Patient outcomes	In June 2020, from 146 home-based patients with spinal cord injury, 6.0% reported a decrease in outpatient care services, 19.2% a decrease for medical treatment by their doctors, 10.0% a decrease for home-visit nursing or rehabilitation, and 4.0% a decrease for home-visit caregiver services due to the pandemic As a result of the pandemic, 40% reported a decrease in health-related quality of life, which included worse pain/discomfort and mobility.
Righi et al. (2022), Out-patient, Italy	To quantify the experienced by adults living with spinal cord injury in Italy during COVID-19 pandemic.	Quantitative Observational (survey)	N, Healthcare access & utilization; Patient experiences	82.2% participants reported deferment or cancellation of non-COVID-19 related health services 13.9% of participants had asked their caregivers to reduce their services; and 12.9% experienced a refusal from their caregivers to guarantee the usual level of assistance
Rispoli & Cappelletto (2022), Out-patient, Italy	To test a teleconsultation procedure suitable for both diagnostic and therapeutic needs of outpatients in our Spine and Spinal Cord Surgery Units.	Quantitative Prospective (intake questionnaire and evaluation of video consults)	Y, Care Delivery	Telemedicine visits had comparable clinical assessments and treatment quality to in-person Patients who used telemedicine were satisfied and would agree to use it in the future Telemedicine is an invaluable tool for safely and timely evaluation and treatment of spinal patients during the pandemic
Rohn et al. (2022), Out-patient, United States	This exploratory online qualitative study collected self-reported COVID-19 experiences from persons with spinal cord injury in the United States (US).	Qualitative (survey)	N, Healthcare access & utilization; Patient experiences	Concerns with health and access to care during pandemic Participants experienced closed clinics, canceled appointments, medical supplies/equipment shortage, lack of access to specialists and delayed procedures Diet, inactivity, and lack of access to a wheelchair specialist resulted in increased pain, potentially lead to long-term health risks and degradation of physical ability Limited access to equipment during the pandemic could lead to increased bladder and bowel management issues Individuals experienced issues with caregiving, especially access to competent and healthy home personal care attendants
Sarkar et al. (2022), In-patient, India	To share experience of providing care for acute spinal disorders during nationwide lockdown and closure of elective services.	Quantitative Prospective Descriptive (chart review)	Y, Healthcare access & utilization; Patient outcomes	During lockdown elective cases were postponed There was an increase incidence in surgical site infections among operated patients (16.7% vs. 3.67%) compared to previous base line
Simpson et al. (2022), Out-patient, Canada	To explore the experiences of individuals with spinal cord injuries during the COVID-19 pandemic.	Qualitative (interview)	N, Care Delivery; Patient experiences	Caregiver support was an issue during the pandemic, with some outlining shortages in home support workers, and others fortunate to maintain caregiver support. Most found telemedicine convenient, but frustrated the service was not offered prior to the pandemic. Participants struggled to understand and apply new information regarding the pandemic
Vives Alvarado et al. (2022), Out-patient, United States	To assess changes in access and psychological status during the COVID-19 pandemic in people with spinal cord injury (SCI).	Quantitative Cohort (survey)	N, Care Delivery; Patient experiences	During the pandemic, 38% reported limited access to personal protective equipment, and 34% reported limited access to medication refills 40% of participants had limited access to healthcare information and services.

### Study design and data source

Eight studies were qualitative, with three using interviews [9, 32, 42], four using surveys with open-ended questions [17, 31, 34, 46], and one using focus groups [41]. Fifteen studies were quantitative, with four that were retrospective using patient chart data or health administrative databases [2, 30, 33, 36, 37], eight using survey data [29, 35, 39, 43, 45, 48–50], and three using patient data (prospective chart review/clinical data, intake questionnaire and video consults) [38, 40, 44]. One study was mixed methods and used survey data which included open-ended and closed-ended questions [47]. Two studies used healthcare provider [47, 50] data and 22 studies reported on patients/individuals with SCI/D data [2, 9, 17, 29–46, 48, 49].

### Participant characteristics

Characteristics of participants of patient and individual level studies are reported in Table 2. Of the 22 studies that used patients/individuals level SCI/D data, 11 studies investigated findings in both TSCI and NTSCD [2, 17, 31–34, 40, 41, 44, 48, 49], and two studies only focused on TSCI [35, 36]. However, nine studies did not specify if participants had a TSCI or NTSCD [9, 29, 30, 38, 39, 42, 43, 45, 46]. Most studies (19/22) reported additional injury/dysfunction and/or comorbidities characteristics of participants [9, 17, 30–36, 38, 39, 41–46, 48, 49]. With the exception of two studies [2, 44], all other studies reported the breakdown of either gender or sex for participants, with no studies reporting both [9, 17, 29–36, 38–43, 45, 46, 48, 49]. No studies investigated differences between sex or gender. Beyond age and sex/gender, twelve studies reported other sociodemographic factors, such as race/ethnicity [9, 17, 31, 32, 34, 35, 41, 42, 45, 46, 48, 49]. However, only one study investigated if patient characteristics impacted healthcare utilization, access, and delivery or patient outcomes and experiences [33]. Kaner et al. (2022) investigated the impact race had on testing positive for COVID-19 [33].

**Table 2:** Individuals with SCI/D sociodemographic characteristics for studies that used patient/individual-level data.

Author (year)	Sample Size	SCI/D Group characteristics				
		Age	Sex/Gender	Reported- Ethnicity/race (Y/N), Additional Sociodemographic (Y/N), Comorbidities (Y/N)	Non-Traumatic (NTSCD) or Traumatic (TSCI)	SCI-injury related characteristics
Bhuva et al. (2020)	680 patients (172 with SCI/D)	Mean (SD): 64.47 (12.42) yrs	**Sex-** Male: 46.7%, Female: 53.3%	Ethnicity/race: N, Sociodemographic: N, Comorbidities: N	NR	NR
D’Andrea et al. (2020)	15 patients with SCI/D; 17 controls	**COVID symptoms** (median (25th–75th percentile)): 60.5 (54.2–70.0) yrs; **No COVID symptoms** (median (25th–75th percentile)): 57.0 (49.0–67.0) yrs.	**Sex-** Male: 67%	Ethnicity/race: N, Sociodemographic: N, Comorbidities: Y	NR	**COVID symptoms:** **Level**- Cervical: 50%, Thoracic: 40%, Lumbosacral: 10%. **Completeness**- Complete motor lesion (AIS A+B): 50%, Incomplete motor lesion (AIS C+D): 50%. **Time since injury (median (25th–75th percentile):** 3.0 (2.0–33.0) yrs **No COVID symptoms:** **Level**- Cervical: 60%, Thoracic: 20%, Lumbosacral: 20%. **Completeness**- Complete motor lesion (AIS A+B): 20%, Incomplete motor lesion (AIS C+D): 80%. **Time since injury median (25th–75th percentile):** 96.0 (12.0–118.0)
Barrows & Goldstein (2021)	Over 25,000 individuals with SCI/D	NR	NR	Ethnicity/race: N, Sociodemographic: N, Comorbidities: N	Both NTSCD and TSCI	NR
Battisti et al. (2021)	501 patients (103 patients with Vertebral Fractures)	Mean (SD): 76.4 (10.8) yrs (for those with Vertebral Fractures)	**Sex**- Male: 60.3% for those with Vertebral Fractures)	Ethnicity/race: N, Sociodemographic: N, Comorbidities: Y	Only TSCI	**Time since injury**: Newly injured
Crawford et al. (2021)	87 patients	Mean age (SE): 59.4 (1.5) yrs (no change in procedure); Mean age (SE): 63.7 (4.5) yrs (change in procedure)	**Sex-** Male: 40.9%, Female: 60.1%	Ethnicity/race: N, Sociodemographic: N, Comorbidities: Y	NR	NR
Hatefi et al. (2021)	11 patients with SCI/D	Mean (SD): 54.2 (9.8) yrs	**Sex-** Male: 68%, Female: 32%	Ethnicity/race: N, Sociodemographic: Y, Comorbidities: N	NR	**Time since injury (mean (SD):** 12.8 (4.9) years
Mikolajczyk et al. (2021)	187 individuals with SCI/D	Mean (SD): 57.0 (14.5) yrs	**Gender-** Men: 73.8%, Women: 25.1%, Transgender/another category: 1.1%	Ethnicity/race: Y, Sociodemographic: Y, Comorbidities: N	TSCI: 86.6% NTSCD: 13.4%	**Level:** Paraplegia: 51.7%, Tetraplegia: 48.3%, Missing: 11. **Completeness:** 32.5%; Incomplete: 67.5%; Missing: 11. **Time since injury (Mean (SD)):** 20.4 (SD 14.9) yrs
Monden et al. (2021)	187 individuals with SCI/D	Mean (SD): 57.0 (14.50) yrs	**Gender-** Men: 73.8%, Women: 25.13%, Transgender or another category: 1.07%	Ethnicity/race: Y, Sociodemographic: N, Comorbidities: N	Traumatic: 86.6% Non-traumatic: 13.4%	**Level-** Paraplegia: 51.71%, Tetraplegia: 48.30%. **Completeness-** Complete: 32.53%, Incomplete: 67.47%.; **Time since injury (Mean (SD)):** 20.37 (14.87) years
Bhattarai et al. (2022)	14 individuals with SCI/D	Mean (SD): 38 (9) yrs	**Sex**- Male: 50%, Female: 50%	Ethnicity/race: Y, Sociodemographic: N, Comorbidities: N	TSCI: 92.9%; NTSCD: 7.1%	**Level-** Cervical: 14.3%, Thoracic: 21.4%, Lumber: 64.3%. **Completeness-** Complete: 85.7%, Incomplete: 14.3%. **Time since injury-** 1–5 yrs: 7.1%, 6–10 yrs: 42.9%, 11–15 yrs: 21.4%, 16–20 yrs: 28.6%
Dean et al. (2022)	12 individuals with SCI/D	Mean (SD): 51.8 (14.5) years	**Sex-** Male: 41.7%, Female: 58.3%	Ethnicity/race: N, Sociodemographic: Y, Comorbidities: N	TSCI: 75% NTSCD: 25%	**Level**- Cervical: 25%, Thoracic: 66.7%, Lumbar: 8.3%. **Completeness:** Motor incomplete: 33.3%, Sensory incomplete: 41.7%, Complete: 25%. **Time since injury-** Mean (SD): 17.9 (13.4) years
Faleiros et al. (2022)	204 individuals with SCI/D	Mean (SD): 40.71 (10.70) yrs	**Sex-** Male: 65.2%, Female: 34.8%	Ethnicity/race: N, Sociodemographic: Y, Comorbidities: N	NR	**Level**- Paraplegia: 62.74%, Tetraplegia: 31.37%, Myelomeningocele: 5.88%.
Hearn et al. (2022)	42 individuals with SCI/D	Mean (SD): 50 (12.5) yrs	**Gender-** Man: 19%, Woman: 81%	Ethnicity/race: Y, Sociodemographic: N, Comorbidities: N	TSI: 52.4%, NTSCD: 38.1%, Prefer not to say: 9.5%	**Level-** Cervical: 26.2%, Thoracic: 40.5%, Lumbar/Sacral: 9.5%; N/A: 16.7%; Not reported: 7.1%. **Completeness-** Complete: 31.0%, Incomplete: 69.0%. **Time since injury:** 1–5 years: 31%, 6–10 years: 14.3%, 11–15 years: 21.4%, 16–20 years: 9.5%, 21+ years: 23.8%
Hill et al (2022)	10 individuals with mid-cervical SCI/D	20-29yrs: 20%, 30-39yrs: 60%, 40-49yrs: 20%	**Sex-** Male: 80%, Female: 20%	Ethnicity/race: Y, Sociodemographic: Y, Comorbidities: N	TSCI: 90%; NTSCD: 10%	**Level:** Mid-cervical: 100%.
Kaner et al. (2022)	4562 individuals with SCI/D	Mean (SD): 65.3 (11.9) yrs	**Gender-** Male: 95%, Female: 5%	Ethnicity/race: N, Sociodemographic: N, Comorbidities: Y	Both NTSCD and TSCI	**Level:** AIS D: 38%; Paraplegia: 22%; Tetraplegia: 19%; Not classified: 22%.
Lakhani et al. (2022)	Timepoint 1: 127 individuals with SCI/D; Timepoint 2: 71 individuals with SCI/D	Mean (SD): 55.81 (12.25)	**Sex-** Male: 74.3%, Female = 25.7%	Ethnicity/race: N, Sociodemographic: Y, Comorbidities: Y	NR	**Level**- Paraplegia: 53.03%, Tetraplegia: 46.97%, **Time since injury (Mean (SD)):** 16.30 (14.07) years
Matsuoka & Sumida (2022)	135 patients with SCI/D	18-20yrs: 1.5%, 20s: 3.0%, 30s: 2.2%, 40s: 5.9%, 50s: 13.3%, 60s: 19.3%, 70s: 34.8%, 80s: 17.8%, 90s: 2.2%	**Sex-** Male: 70.9%, Female: 29.1%	Ethnicity/race: N, Sociodemographic: N, Comorbidities: N	NR	**Level-** Cervical: 70.5%, Thoracic/lumbar: 25.0%, Other: 4.5%. **Completeness:**-Complete: 95.5%, Unknown: 4.5%; **Time since injury-** 1y: 10.4%, 2y: 13.7%, 3y: 13.7%, 4y: 18.3%, 5y: 13.7%, 6y: 6.9%, 7y: 5.3%, 8y: 6.9%, 9y: 2.3%, 10y and over 11: 8.4%
Righi et al. (2022)	101 individuals with SCI/D	Median: 53 yrs (interquartile range: 47–61)	**Sex-** Male: 66.3%; Female: 33.7%	Ethnicity/race: N, Sociodemographic: N, Comorbidities: Y	NR	**Level-** Paraplegic: 56.4%. **Completeness**: Motor complete: 53.4%.
Rispoli & Cappelletto (2022)	50 individuals with SCI/D	second study only:-Mean: 51 (range = 18–96)	**Gender** (second study only)- Male: 37.1%, Female:62.9%	Ethnicity/race: N, Sociodemographic: N, Comorbidities: N	TSCI (vertebral fractures): 51.4%, NTSCD (degenerative/ other): 11.4%, Unclear (postoperative follow-up): 37.1%.	NR
Rohn et al. (2022)	36 individuals with SCI/D	Mean (SD): 55.7 (13.1) yrs	**Gender-** Men: 75%, Women: 25%	Ethnicity/race: Y, Sociodemographic: N, Comorbidities: N	TSCI: 63.9%, NTSCD: 11.1%, Other = 25.0%	**Level-** Cervical: 50.0%, Thoracic = 41.7%, Lumbar/Sacral = 2.8%, N/A (degenerative disease) = 5.6%. **Completeness-** Complete: 94.4%, Incomplete = 5.6%. **Time since injury (Mean (SD)):** 21.5 (13.6) yrs
Sarkar et al. (2022)	24 patients with spinal conditions	NR	NR	Ethnicity/race: N, Sociodemographic: N, Comorbidities: N	TSCI: 79.2%, NTSCD (Infection & Tumor): 20.8%	**Level:** Cervical: 12.5%, Dorso-lumbar: 83.3%, Sacral: 4.2%.
Simpson et al. (2022)	22 individuals with SCI/D	Mean (SD): 53.77 (11.07) yrs	**Sex-** Male: 55%, Female: 41%, Not-disclosed: 5%	Ethnicity/race: N, Sociodemographic: Y, Comorbidities: N	NR	**Level:** Paraplegia: 45%, Tetraplegia: 27%, Other: 27%. **Completeness:** Complete: 7%, Incomplete: 14%, Not answered: 5%. **Time since injury:** Since birth:9%, Since childhood: 5%, Since adolescence: 18%, Adulthood: 27%, Later in life: 41%
Vives Alvarado et al. (2022)	51 individuals with TSCI	NR	**Sex-** Male: 94%, Female: 6%	Ethnicity/race: Y, Sociodemographic: N, Comorbidities: N	TSCI	**Level:** Paraplegia: 39%, Tetraplegia: 47%, Unknown: 14%. **Time since injury:** 1–2 years: 51%, 5–6 years: 35%, 15–16 years: 14%

### Overview of key findings

Eleven studies primary purpose, objectives and findings were only healthcare-related topics during the pandemic [2, 29–31, 33, 36, 38, 40, 44, 47, 50]. Of these studies, three focused on the outcomes of the COVID-19 infection in individuals with SCI/D [33, 36, 38], five focused on virtual care [2, 29–31, 40], two focused on the SCI/D healthcare communities response to the pandemic from the perspective of healthcare providers [47, 50], and one study focused on providing in-patient care for acute spinal disorders during the pandemic [44]. An additional three studies had both health-related and non-health related objectives and findings [45, 48, 49]; whereas eight studies’ objectives focused on general perceptions, experiences, challenges, and impact the COVID-19 pandemic had on individuals with SCI/D, but their findings included healthcare-related topics [9, 17, 32, 34, 39, 41, 42, 45, 46]. Two studies had objectives focused on changes in mental health /quality of life status, but also had healthcare-related findings [35, 43].

#### Healthcare access and/or utilization

Sixteen studies had findings related to healthcare utilization and/or access [9, 17, 32, 34, 35, 39, 41–43, 45–50]. However only one study’s main objective was healthcare-related [47]. With the pandemic onset, access to healthcare related services for individuals with SCI/D was consistently found to be challenging, with this finding spanning multiple countries and healthcare systems [17, 34, 35, 41–43, 45, 46, 49]. In the outpatient settings challenges included decreased access to specialists, medical supplies/equipment, medication, therapies, outpatient services, and homecare services, as well as delays or cancellation of appointments and medical procedures [17, 34, 35, 41–43, 45, 46, 49]. Matsuoka & Sumida (2022) was the only study to investigate homecare utilization and access, and found home-based individuals with SCI/D reported a 10.0% decrease for home-visit nursing or rehabilitation, and a 4.0% decrease for home-visit caregiver services during the pandemic [43]. For the in-patient setting elective services were postponed [44]. Issues with access to healthcare was found to negatively impact overall and mental health [48]. Only one study investigated access to testing and screening of COVID-19 for individuals with SCI/D [50]. Stillmen et al. found testing and screening of the COVID-19 virus varied substantially across countries and health systems based on availability of testing kits and guidelines/protocols [50].

#### Care delivery

With the onset of the pandemic, the use of virtual care as a delivery format rose rapidly in the SCI/D population, whereas prior to the pandemic it was a less frequent delivery format [2, 9, 47]. Eight studies identified findings related to virtual care [2, 17, 29–32, 40, 47], with five of these studies primary purpose to investigate virtual care utility, experience and/or delivery in the SCI/D population during the pandemic [2, 29–31, 40]. Virtual care was used to align with government safety guidelines, while ensuring continued patient access, continuity of care and patient and provider safety [2, 30, 32, 40, 47]. The delivery of virtual care was mainly video-based [2, 29, 30], with less than 10% only using audio [29, 30]. There were many reported advantages to using virtual care [2, 17, 29–32, 40, 47]. Patients with SCI/D noted scheduling routine appointments was easier with virtual care [31]. The use of virtual care was reported to reduce the transportation burden and associated costs [31, 32]. Patients reported virtual care was a convenient method for accessing prescription refills, consulting on current medications, patient education, various cognitive therapies and follow-ups with physicians [2, 9, 31, 32, 40]. Studies also reported a high-level of patient satisfaction with virtual care use [29, 40], and similar levels of quality and/or treatment plans as in-person visits [30, 40]. The studies reported patients may prefer the use virtual care services due these advantages, as well as helping to prevent COVID-19 exposure and maintaining social distancing [29, 31, 32, 40]. However, three studies did report that virtual care as a delivery format had limitations with physical assessments and was not favourable for certain physical treatments, such as physiotherapy or injections [2, 31, 32]. One qualitative study reported patients found building a rapport with their providers through virtual care was challenging (e.g., impersonal and rushed) [31]. Furthermore, four studies outlined other concerns over the implementation and sustained use of virtual care for the SCI/D population [29, 31, 32, 47]. Virtual care may not be accessible to all patients and providers, as it requires access to technology and appropriate digital literacy [31, 32, 47]. There is also a possibility of technology-related issues, such as poor audio or video, during virtual care appointments [29, 31]. Privacy and confidentiality concerns with telemedicine use that need to be addressed before further implementation and expansion were also described [31, 47]. Finally, six studies found either patients or healthcare providers agreed that virtual care delivery was a useful tool in their care routines, but could not completely replace in-person care healthcare delivery [2, 17, 29, 31, 40, 47].

Beyond investigating virtual care, only one study, an international quantitative survey with healthcare providers, investigated care delivery for individuals with SCI/D who tested positive for COVID-19 [50]. It was also the only study to investigate care delivery in the in-patient setting [50].

#### Patient outcomes

A total of nine studies investigated patient outcomes during the pandemic [33, 36–38, 41, 43–45, 47]. Of these, four studies had findings related to COVID-related health outcomes [33, 36, 38, 41]. When infected with the COVID-19 virus, two studies found no significant difference between a control group and individuals with SCI/D clinical outcomes, including mortality [36, 38]. Specifically, one study found no significant differences in COVID-19 symptoms and treatment between the control group and individuals with SCI/D, with no deaths or treatment in intensive care units for either group [38]. The other study found having a SCI/D in the form of a vertebral fracture was not associated with mortality in patients with COVID-19, compared to those without a vertebral fracture and COVID-19 [36]. However, one study found other patient characteristics, such as age, race, comorbidities, body mass index and smoking status may be associated with increased risk to testing positive for the virus and/or mortality when infected [33]. A qualitative focus group study found that some individuals with SCI/D, who contracted the COVID-19 virus, had symptoms that severely impacted their lives and resulted in hospitalization [41].

Six studies had findings related to general health outcomes during the pandemic [37, 41, 43–45, 47]. During the pandemic, three survey studies and one focus group study reported individuals with SCI/D had increased or worsened secondary conditions, such as urinary tract infections, pressure ulcers, and pain [41, 43, 45, 47]. A quantitative study using prospective patient data in an in-patient setting, found 2/12 (16.7%) operated patients experienced surgical site infections, an increase from the previous baseline of 8/218 (3.7%) operated patients [44]. A qualitative open-ended survey found overall individuals with SCI/D felt they experienced a deterioration in physical health and well-being during the pandemic [17].

#### Patient experiences

Ten studies investigated topics related to patient experiences during the COVID-19 pandemic among individuals with SCI/D [9, 17, 32, 34, 35, 39, 41, 42, 47, 49], with five studies exploring caregiving support [32, 34, 39, 41, 47], six studies exploring issues accessing medical supplies, and four studies exploring COVID-19 related health literacy [9, 35, 42]. All five studies on caregiving reported that individuals with SCI/D experienced changes to caregiving support during the pandemic [32, 34, 39, 41, 47]. It was difficult to maintain caregiving at home due to shortages of competent and healthy personal care workers [9, 34, 39, 41, 47]. There were concerns of increased exposure risk with formal caregivers who worked with multiple clients, leading some to stop using external caregiving services temporarily [32, 39]. One qualitative study found due to these concerns, individuals described they had a decrease in formal caregiving, leading them to rely more on informal caregivers and ultimately resulting in increased demand and stress for their families [32]. However, two studies, one qualitative interview with patients and another quantitative survey, found some individuals were able to maintain usual/adequate caregiving services support during the pandemic [9, 39]. Righi et al. (2022), found in 101 individuals, 13.9% of participants had asked their caregivers to reduce their services and 12.9%, experienced a refusal from their caregivers to guarantee the usual level of assistance [39].

Six studies outlined individuals with SCI/D experienced issues with medical device and supplies shortages [17, 32, 34, 35, 41, 49]. Four qualitative studies outlined that participants felt they had inadequate access to medical resources, supplies and equipment [17, 32, 34, 41]. One quantitative study reported over 38% or individuals reported limited access to personal protective equipment [35], and another quantitative study reported 59% of participants reported a negatively impacted to medical supplies access due to the pandemic [49].

Three studies with findings related to health literacy, found individuals with SCI/D felt they needed better access to information related to the pandemic and the COVID-19 virus [9, 35, 42]. Individuals with SCI/D struggled to stay up-to-date with the evolving restrictions, guidelines, and information/misinformation surrounding the COVID-19 virus and pandemic [9, 42]. They expressed having minimal understanding of the COVID-19 virus and were unsure of how to access appropriate and accurate information [9, 35, 42]. Similarly, Gustafson et al. (2021) reported over 60% of healthcare providers also felt more education is needed about how COVID-19 affects people with disabilities and how COVID-19 can impact patients’ rehabilitation [47].

## Discussion

The purpose of our scoping review was to identify literature pertaining to healthcare and healthcare-related experiences and outcomes of individuals with SCI/D due to the impact of the COVID-19 virus and pandemic. Considering the broad scope of the scoping review, overall, we found surprisingly minimal research on this topic. Although we found 24 articles, only 11 focused on healthcare related topics [2, 29–31, 33, 36, 38, 40, 44, 47, 50], with the remaining 13 articles exploring general experiences of the SCI/D population during the pandemic [9, 17, 32, 34, 35, 39, 41–43, 45, 46, 48, 49]. As such these 13 articles provided only minimal information about healthcare and healthcare-related outcomes of individuals with SCI/D due to COVID-19. We also found a range in study designs which included quantitative [2, 29, 30, 33, 35, 36, 38–40, 43, 44, 48–50], qualitative [9, 17, 31, 32, 34, 41, 42, 46], and mixed-methods [47]. However, half of the qualitative studies were open-ended survey designs [17, 31, 34, 46], therefore providing limited understanding and exploration of patient experiences. No studies quantified changes in healthcare utilization, access, delivery, or outcomes. It was found virtual care had many advantages and was a good avenue to maintain continuity of care for the SCI/D population during the pandemic; however, was not suitable for all forms of care [2, 17, 29–32, 40, 47]. The SCI/D population also experienced changes in usual caregiving support during the pandemic [32, 34, 39, 41, 47]. Since there was limited investigation on the impact the COVID-19 virus on the SCI/D population, it was unclear if the virus impacted individuals with SCI/D differently [36, 38].

Fifteen studies had healthcare utilization and access related findings, however no studies’ primary purpose or objective was to investigate shifts in healthcare utilization and/or access in the SCI/D population during the pandemic [9, 17, 32, 34, 35, 39, 41–43, 45–49]. Although it is clear that based on the findings from the scoping review individuals with SCI/D experienced decreased access and utilization of health-related services during the pandemic, it is unclear to what extent access decreased and the longer-term impacts decreased services access [9, 17, 32, 34, 35, 39, 41–43, 45–49]. None of the studies quantified changes in healthcare utilization or access, with most studies investigating this concept being qualitative. Consequently, the findings related to healthcare utilization and access were mainly from patient perspectives. Even within the qualitative studies, there was no in-depth investigation of barriers and facilitators to healthcare utilization or access, as four of the eight qualitative studies used open-ended surveys [9, 17, 31, 32, 34, 41, 42, 46]. The lack of information on the barriers and facilitators to healthcare utilization may negatively impact policy development and healthcare planning. Quantifying shifts in healthcare utilization and access can help with determining resource allocation and associated cost, identifying gaps in healthcare provision, as well as health system planning [51]. Investigating and quantifying these changes during the pandemic for the SCI/D population can help healthcare providers understand how the pandemic has affected their patient populations’ access to care. By identifying these shifts, healthcare providers and policymakers can take the needed steps to develop interventions, guidelines, resources, or policies to ensure that healthcare services are accessible and safe for the SCI/D population.

Virtual care was seen as a tool to maintain access and continuity of care in the SCI/D population [2, 17, 29–32, 40, 47]. Findings from our scoping review suggest that virtual care was well-received and accepted by individuals with SCI/D due to its many advantages [2, 17, 29–32, 40, 47]. However, there are certain types of care, which involve physical assessments, that cannot be conducted virtually, such as hence in-person visits are still needed [2, 31, 32]. As such, a blended model of both virtual and in-person care delivery may be most beneficial to the SCI/D population [31]. This finding is in line with a recent systematic review in the general population, which found a hybrid model of in-person and virtual care would be idea [14]. This systematic review highlighted a substantial amount of research on virtual care during the pandemic internationally, including how virtual visits helped maintain access and care during the pandemic, but also conceded virtual care could not replace in-person visits [14].

Issues with maintaining caregiving support was found to be a key component of patient experiences for individuals with SCI/D [32, 34, 39, 41, 47]. These findings were mainly from qualitative studies, and relied on patient perspectives [32, 34, 41, 47]. Only one study, by Righi et al. (2022) attempted to quantify changes in caregiving [39]. Furthermore, Matsuoka & Sumida (2022) was the only study to investigate homecare utilization and access [43]. It should be noted both studies were quantitative surveys and neither study’s primary purpose was focused on healthcare, and provided limited findings regarding caregiving and homecare utilization [39, 43]. It is well established that caregiving support and homecare services are a vital component to helping individuals with SCI/D remain safely in the community setting [52]. To understand the SCI/D population care in the community during the pandemic, it is important to have in-depth investigation of shifts in caregiving support and homecare services, and to quantify any changes. Current literature has insufficient findings regarding the current state making it difficult to understand or map shifts in caregiving and homecare service utilization during the pandemic for the SCI/D population.

During the pandemic individuals with SCI/D residing in the community reported experiencing increases in secondary complications and poor health outcomes across different countries and health care systems [17, 32, 41, 43, 45, 47]. Individuals with SCI/D may not be getting their healthcare needs met during the pandemic, due to decreases in healthcare utilization and access, as well as shifts in usual caregiving support [9, 17, 32, 34, 35, 39, 41–43, 45–49]. However, it should be noted these findings are based on qualitative studies that reported on patient perspectives, and currently there are no quantitative population-based studies investigating secondary complications or patient outcomes, including mortality, during the pandemic.

There was limited research on patient outcomes related to COVID-19 virus infection. Only two studies compared a control group to individuals with SCI/D when infected with the COVID-19 virus, and found no significant differences between to the two groups [36, 38]. However, a qualitative study with individuals with SCI/D, who contracted the COVID-19 virus, reported their associated symptoms severely impacted their lives and resulted in hospitalization [41]. A previous rapid review was conducted to evaluate the COVID-19 virus in individuals with SCI/D, and included 10 studies, which consisted of a case-control series, a case series, four case reports, two editorial pieces, a cross-sectional registry study, and cross-sectional survey study with healthcare professionals [53]. The rapid review found the clinical presentation of the COVID-19 virus in individuals with SCI/D was comparable to the general population, however rates of mortality rate maybe higher in individuals with SCI/D [53]. Although this rapid review provided insight into the COVID-19 virus presentation in SCI/D, no reliable conclusions can be made due to the small number of patients in the included studies, which were mainly observational [53]. Based on the findings from these limited studies investigating the COVID-19 virus clinical manifestation and outcomes in our review, as well as this previously conducted rapid review, it is still unclear if the COVID-19 virus impacts individuals with SCI/D differently or more severely. This lack of clarity, may be the reason why individuals with SCI/D felt they had insufficient information related to the COVID-19 pandemic and virus [9, 35, 42]. This sentiment was echoed by healthcare providers as Gustafson et al. (2021) reported they also felt more education is needed about how COVID-19 effects people with disabilities and how COVID-19 impacts patients’ rehabilitation [47]. In order to address the concerns of healthcare providers and individuals with SCI/D lack of awareness, education and knowledge about the COVID-19 virus impact on individuals with SCI/D, more research is needed in this area.

### Gaps and opportunities for future research

Overall, this scoping review highlighted more research is needed to investigate the impact the COVID-19 pandemic had on individuals with SCI/D with regards to healthcare access, utilization, and delivery, as well as patient outcomes and experiences. Here are our recommendations for future research based on identified gaps:

1. Currently, there is limited understanding on how the COVID-19 virus impacts individuals with SCI/D. Further research is needed to investigate the short and long-term outcomes of individuals with SCI/D when infected with the COVID-19 virus.
2. Only one study investigated differences between race/ethnicity [33]. Kaner et al. investigated the impact race had on testing positive for COVID-19 [33]. However, there has been no investigation on the impact race and/or ethnicity had on shifts in healthcare utilization, access, delivery, and outcomes. Research should investigate the impact race and/or ethnicity had on healthcare in individuals with SCI/D.
3. Research is needed to investigate how the pandemic affected different sexes, gender, socioeconomic groups, ages, or injury-related characteristics, such as NTSCD versus TSCI. It is important to investigate differences between these patient groups within the SCI/D population, as it is possible certain groups experiencing a greater reduction in care or negative impacts due to the pandemic [54]. By identifying if such differences exist, interventions can be created to target the most vulnerable groups to improve their care.
4. No studies quantifying changes to healthcare utilization, healthcare access, caregiving support, homecare services, uptake of virtual care, and patient outcomes (secondary conditions and mortality) in the SCI/D population during the pandemic. Future studies should investigate the impact of the pandemic on primary care, specialist care, homecare, emergency department visits, and hospital admissions utilization and delivery. Large-scale quantitative studies investigating these topics are needed to inform policy development and healthcare planning for the SCI/D population.
5. Only three studies investigated the in-patient setting, as such more research is needed to understand how the pandemic impacted the in-patient SCI/D population.
6. Research would also benefit from understanding the perspective of a variety of interest groups, and future research should include collaboration with healthcare providers and organizations.
7. Future studies should continue to investigate and improve patient experiences, population health, provider experience, health equity and healthcare value in the SCI/D population in a post-pandemic period as well.

### Limitations

This scoping review had a few limitations. It is possible that relevant articles were missed as we excluded opinion pieces, conference abstracts, study protocols, quantitative case studies (less than 10 individuals) and articles in which we could not access the full-text through our library or interlibrary loan system. However, the University of Toronto has an extensive catalogue of resources, and is the largest academic library in Canada, and only 6 full-text articles could not be accessed. Our search strategy included both individuals with TSCI and NTSCD, however terms associated with NTSCD are not well defined making it difficult to identify or include studies investigating NTSCD [23].

## Conclusion

This scoping review identified the type and extent of literature pertaining to the health-related impact the COVID-19 pandemic and virus had on the SCI/D population. Although scope of this review was quite broad, we found limited research that focused on the healthcare impacts of the COVID-19 pandemic and were able to identify key areas that require further investigation. More research is needed to understand how the COVID-19 virus infection impacts individuals with SCI/D as there have been only two studies investigating this topic, with these findings needing to be disseminated to healthcare providers as well as individuals with SCI/D. Further investigation, especially quantitative studies, are needed to identify the impact the pandemic had on the SCI/D population health outcomes across different patient impairment groups or subgroups to better inform policy development and healthcare planning.

## Supporting information

S1 Appendix Search strategy. Contains full search strategy for all databases used for scoping review.
(DOCX)
