# Supplementary material for: The impact of the COVID-19 virus and pandemic on healthcare utilization, access, delivery, experiences, and outcomes in the spinal cord injuries/dysfunction population: A scoping review study
Source: PLoS One. 2024 Feb 22;19(2):e0297384. doi: 10.1371/journal.pone.0297384 (PMC10883570; doi:10.1371/journal.pone.0297384)
Supplement: S1 Appendix — Contains full search strategy for all databases used for scoping review. (DOCX) [file pone.0297384.s001.docx]

**Appendix 1- Search Strategy**

**MEDLINE**

| **#** | **Search Term** |
| --- | --- |
| 1 | exp Spinal Cord/in [injuries] |
| 2 | exp Central Cord Syndrome/ |
| 3 | exp Cervical Vertebrae/in [injuries] |
| 4 | spinal cord compression/ or spinal cord injuries/ or central cord syndrome/ or spinal injuries/ or spinal fractures/ or Spinal Cord Ischemia/ |
| 5 | spinal cord diseases/ or amyotrophic lateral sclerosis/ or epidural abscess/ or muscular atrophy, spinal/ or myelitis/ or spinal cord compression/ or spinal cord injuries/ or spinal cord neoplasms/ or spinal cord vascular diseases/ or spinocerebellar degenerations/ or subacute combined degeneration/ or syringomyelia/ or tabes dorsalis/ |
| 6 | spinal diseases/ or intervertebral disc degeneration/ or intervertebral disc displacement/ or posterior cervical sympathetic syndrome/ or spinal neoplasms/ or spinal osteochondrosis/ or spinal osteophytosis/ or spinal stenosis/ or spondylitis/ or spondylosis/ |
| 7 | myelitis, transverse/ or paraparesis, tropical spastic/ or epidural neoplasms/ or anterior spinal artery syndrome/ or spinal cord ischemia/ or degenerative disc disease/ |
| 8 | ((spine* or spinal* or vertebr* or lumbar or central cord) adj4 (injur* or non-trauma* or nontrauma* or contusion* or dysfunct* or laceration* or trauma* or transection* or damag* or fractur* or compress* or weak?n* or atroph* or ischemi* or syndrome* or break* or broke* or dissect* or lesion* or h?emorrhag* or neoplasm* or disease* or myelopath* or myelitis or wound or degenerat* or tumo?r )).tw,kf. |
| 9 | (fractur* adj3 (burst or chance or compression* or lumbar* or sacrum* or thoracic)).tw,kf. |
| 10 | (trauma* adj3 myelopath*).tw,kf. |
| 11 | (hangman* adj2 fractur*).tw,kf. |
| 12 | Paraplegia/ or quadriplegia/ or hemiplegia/ or paraparesis/ or paresis/ |
| 13 | (Paraplegi* or quadriplegi* or tetraplegi* or hemiplegi* or SCI or paraparesis or paresis or paraly?ed or quadripares* or syringomyelia).tw,kf |
| 14 | 1 OR 2 OR 3 OR 4 OR 5 OR 6 OR 7 OR 8 OR 9 OR 10 12 OR 13 |
| 15 | COVID-19/ or exp COVID-19 Testing/ or COVID-19 Vaccines/ or SARS-CoV-2/ |
| 16 | (coronavirus/ or betacoronavirus/ or coronavirus infections/) and (disease outbreaks/ or epidemics/ or pandemics/) |
| 17 | (nCoV* or 2019nCoV or 19nCoV or COVID19* or COVID or SARS-COV-2 or SARSCOV-2 or SARS-COV2 or SARSCOV2 or SARS coronavirus 2 or Severe Acute Respiratory Syndrome Coronavirus 2 or Severe Acute Respiratory Syndrome Corona Virus 2).ti,ab,kf,nm,ot,ox,rx,px. |
| 18 | ((new or novel or 19 or 2019 or Wuhan or Hubei or China or Chinese) adj3 (coronavirus* or corona virus* or betacoronavirus* or CoV or HCoV)).ti,ab,kf,ot. |
| 19 | (longCOVID* or postCOVID* or postcoronavirus* or postSARS*).ti,ab,kf,ot. |
| 20 | ((coronavirus* or corona virus* or betacoronavirus*) adj3 (pandemic* or epidemic* or outbreak* or crisis)).ti,ab,kf,ot. |
| 21 | ((Wuhan or Hubei) adj5 pneumonia).ti,ab,kf,ot. |
| 22 | 15 OR 16 OR 17 OR 18 or 19 or 20 or 21 |
| 23 | 14 AND 22 |

**Embase Classic+ Embase <1947 to 2022 November 10>**

1 amyotrophic lateral sclerosis/

2 central cord syndrome/

3 cervical spinal cord injury/

4 cervical spine injury/

5 epidural abscess/

6 hemiplegia/

7 intervertebral disk degeneration/

8 intervertebral disk hernia/

9 myelitis/

10 paraplegia/

11 paresis/

12 quadriplegia/

13 spinal cord compression/

14 spinal cord injury/

15 spine disease/

16 spine injury/

17 exp spine fracture/

18 spinal cord transsection/

19 spinal cord ischemia/

20 spinal cord disease/

21 spinal muscular atrophy/

22 spinal cord tumor/

23 spinal cord vascular disease/

24 spinocerebellar degeneration/

25 subacute combined degeneration/

26 syringomyelia/

27 spine disease/

28 spondylosis/

29 spine tumor/

30 Scheuermann disease/

31 spondylosis/

32 spondylitis/

33 spondylosis/

34 spinal cord tumor/

35 spinal cord ischemia/

36 spinal cord ischemia/

37 tabes dorsalis/

38 tropical spastic paraparesis/

39 transverse myelitis/

40 vertebral canal stenosis/

41 vertebra dislocation/

42 vertebra compression/

43 ((spine* or spinal* or vertebr* or lumbar or central cord) adj4 (injur* or non-trauma* or nontrauma* or contusion* or dysfunct* or laceration* or trauma* or transection* or damag* or fractur* or compress* or weak?n* or atroph* or ischemi* or syndrome* or break* or broke* or dissect* or lesion* or h?emorrhag* or neoplasm* or disease* or myelopath* or myelitis or wound or degenerat* or tumo?r)).tw,kf.

44 (fractur* adj3 (burst or chance or compression* or lumbar* or sacrum* or thoracic)).tw,kf.

45 (trauma* adj3 myelopath*).tw,kf.

46 (hangman* adj2 fractur*).tw,kf.

47 (paraplegi* or quadriplegi* or tetraplegi* or hemiplegi* or SCI or paraparesis or paresis or paraly?ed or quadripares* or syringomyelia).tw,kf.

48 or/1-47

49 sars-related coronavirus/

50 (coronavirinae/ or betacoronavirus/ or coronavirus infection/) and (epidemic/ or pandemic/)

51 (nCoV* or 2019nCoV or 19nCoV or COVID19* or COVID or SARS-COV-2 or SARSCOV-2 or SARS-COV2 or SARSCOV2 or "SARS coronavirus 2" or "Severe Acute Respiratory Syndrome Coronavirus 2" or "Severe Acute Respiratory Syndrome Corona Virus 2").ti,ab,kw,hw,ot.

52 ((new or novel or "19" or "2019" or Wuhan or Hubei or China or Chinese) adj3 (coronavirus* or "corona virus*" or betacoronavirus* or CoV or HCoV)).ti,ab,kw,hw,ot.

53 (longCOVID* or postCOVID* or postcoronavirus* or postSARS*).ti,ab,kw,hw,ot.

54 ((coronavirus* or "corona virus*" or betacoronavirus*) adj3 (pandemic* or epidemic* or outbreak* or crisis)).ti,ab,kw,ot.

55 ((Wuhan or Hubei) adj5 pneumonia).ti,ab,kw,ot.

56 or/49-55

57 48 and 56

**SCOPUS**

( ( KEY ( coronavirus OR betacoronavirus OR "coronavirus infections" ) AND KEY ( "disease outbreaks" OR epidemics OR pandemics ) ) OR ( TITLE-ABS-KEY ( ncov* OR 2019ncov OR 19ncov OR covid19* OR covid OR sars-cov-2 OR sars-cov2 OR sarscov-2 OR sarscov2 OR "SARS coronavirus 2" OR "Severe Acute Respiratory Syndrome Coronavirus 2" OR "Severe Acute Respiratory Syndrome Corona Virus 2" ) ) OR ( TITLE-ABS-KEY ( ( new W/3 coronavirus* ) OR ( new W/3 "corona virus*" ) OR ( new W/3 betacoronavirus* ) OR ( new W/3 cov ) OR ( new W/3 hcov ) OR ( novel W/3 coronavirus* ) OR ( novel W/3 "corona virus*" ) OR ( novel W/3 betacoronavirus* ) OR ( novel W/3 cov ) OR ( novel W/3 hcov ) OR ( 19 W/3 coronavirus* ) OR ( 19 W/3 "corona virus*" ) OR ( 19 W/3 betacoronavirus* ) OR ( 19 W/3 cov ) OR ( 19 W/3 hcov ) OR ( 2019 W/3 coronavirus* ) OR ( 2019 W/3 "corona virus*" ) OR ( 2019 W/3 betacoronavirus* ) OR ( 2019 W/3 cov ) OR ( 2019 W/3 hcov ) OR ( wuhan W/3 coronavirus* ) OR ( wuhan W/3 "corona virus*" ) OR ( wuhan W/3 betacoronavirus* ) OR ( wuhan W/3 cov ) OR ( wuhan W/3 hcov ) OR ( hubei W/3 coronavirus* ) OR ( hubei W/3 "corona virus*" ) OR ( hubei W/3 betacoronavirus* ) OR ( hubei W/3 cov ) OR ( hubei W/3 hcov ) OR ( china W/3 coronavirus* ) OR ( china W/3 "corona virus*" ) OR ( china W/3 betacoronavirus* ) OR ( china W/3 cov ) OR ( china W/3 hcov ) OR ( chinese W/3 coronavirus* ) OR ( chinese W/3 "corona virus*" ) OR ( chinese W/3 betacoronavirus* ) OR ( chinese W/3 cov ) OR ( chinese W/3 hcov ) ) ) OR ( TITLE-ABS-KEY ( longcovid* OR postcovid* OR postcoronavirus* OR postsars* ) ) OR ( TITLE-ABS-KEY ( ( coronavirus* W/3 pandemic* ) OR ( coronavirus* W/3 epidemic* ) OR ( coronavirus* W/3 outbreak* ) OR ( coronavirus* W/3 crisis ) OR ( "corona virus*" W/3 pandemic* ) OR ( "corona virus*" W/3 epidemic* ) OR ( "corona virus*" W/3 outbreak* ) OR ( "corona virus*" W/3 crisis ) OR ( betacoronavirus* W/3 pandemic* ) OR ( betacoronavirus* W/3 epidemic* ) OR ( betacoronavirus* W/3 outbreak* ) OR ( betacoronavirus* W/3 crisis ) ) ) OR ( TITLE-ABS-KEY ( ( wuhan W/5 pneumonia ) OR ( hubei W/5 pneumonia ) ) ) ) AND ( TITLE-ABS-KEY ( ( "trauma* myelopath*" OR "post-trauma* myelopath*" OR "Paraplegi*" OR "quadriplegi*" OR "tetraplegi*" OR "syringomyelia" OR "degenerative disc disease" OR "epidural abscess" OR "spondylitis" OR "spondylosis" OR "intervertebral disc degeneration" OR "amyotrophic lateral sclerosis" OR "myelitis" OR "spinocerebellar degenerations" OR "subacute combined degeneration" OR "tabes dorsalis" OR "intervertebral disc displacement" OR "posterior cervical sympathetic syndrome" OR "anterior spinal artery syndrome" ) OR ( spinal* W/2 injur* OR non-trauma* OR nontrauma* OR contusion* OR dysfunct* OR laceration* OR trauma* OR transection* OR damag* OR fractur* OR compress* OR weak?n* OR atroph* OR ischemi* OR syndrome* OR break* OR broke* OR dissect* OR lesion* OR h?emorrhag* OR neoplasm* OR disease* OR myelopath* OR myelitis OR wound OR degenerat* OR tumo?r ) OR ( spine* W/3 injur* OR non-trauma* OR nontrauma* OR contusion* OR dysfunct* OR laceration* OR trauma* OR transection* OR damag* OR fractur* OR compress* OR weak?n* OR atroph* OR ischemi* OR syndrome* OR break* OR broke* OR dissect* OR lesion* OR h?emorrhag* OR neoplasm* OR disease* OR myelopath* OR myelitis OR wound OR degenerat* OR tumo?r ) OR ( vertebr* W/2 injur* OR non-trauma* OR nontrauma* OR contusion* OR dysfunct* OR laceration* OR trauma* OR transection* OR damag* OR fractur* OR compress* OR weak?n* OR atroph* OR ischemi* OR syndrome* OR break* OR broke* OR dissect* OR lesion* OR h?emorrhag* OR neoplasm* OR disease* OR myelopath* OR myelitis OR wound OR degenerat* OR tumo?r ) OR ( lumbar W/2 injur* OR non-trauma* OR nontrauma* OR contusion* OR dysfunct* OR laceration* OR trauma* OR transection* OR damag* OR fractur* OR compress* OR weak?n* OR atroph* OR ischemi* OR syndrome* OR break* OR broke* OR dissect* OR lesion* OR h?emorrhag* OR neoplasm* OR disease* OR myelopath* OR myelitis OR wound OR degenerat* OR tumo?r ) OR ( "central cord" W/2 injur* OR non-trauma* OR nontrauma* OR contusion* OR dysfunct* OR laceration* OR trauma* OR transection* OR damag* OR fractur* OR compress* OR weak?n* OR atroph* OR ischemi* OR syndrome* OR break* OR broke* OR dissect* OR lesion* OR h?emorrhag* OR neoplasm* OR disease* OR myelopath* OR myelitis OR wound OR degenerat* OR tumo?r ) OR ( fractur* W/2 burst OR chance OR compression* OR lumber* OR sacrum* OR thoracic ) OR ( hangman* W/2 fractur* ) ) )

**CINAHL**

(MH "Spinal Cord+/IN")

(MH "Central Cord Syndrome")

(MH "Cervical Vertebrae+/IN")

(MH "Spinal Cord Compression")

(MH "Spinal Cord Injuries")

(MH "Spinal Injuries")

(MH "Spinal Fractures")

(MH "Spinal Cord Diseases")

(MH "Amyotrophic Lateral Sclerosis")

(MH "Epidural Abscess")

(MH "Muscular Atrophy, Spinal")

(MH "Myelitis")

(MH "Spinal Cord Neoplasms")

(MH "Spinocerebellar Degenerations")

(MH "Syringomyelia")

(MH "Neurosyphilis")

(MH "Spinal Diseases")

(MH "Intervertebral Disk Displacement")

(MH "Spinal Neoplasms")

(MH "Spinal Cord Neoplasms")

(MH "Spinal Osteophytosis")

(MH "Spinal Stenosis")

(MH "Spondylitis, Ankylosing")

(MH "Spondylosis")

(MH "Myelitis, Transverse")

OR

TI ((spine* or spinal* or vertebr* or lumbar or "central cord") N4 (injur* or non-trauma* or nontrauma* or contusion* or dysfunct* or laceration* or trauma* or transection* or damag* or fractur* or compress* or weak#n* or atroph* or ischemi* or syndrome* or break* or broke* or dissect* or lesion* or h#emorrhag* or neoplasm* or disease* or myelopath* or myelitis or wound or degenerat* or tumo#r))

OR

AB ((spine* or spinal* or vertebr* or lumbar or "central cord") N4 (injur* or non-trauma* or nontrauma* or contusion* or dysfunct* or laceration* or trauma* or transection* or damag* or fractur* or compress* or weak#n* or atroph* or ischemi* or syndrome* or break* or broke* or dissect* or lesion* or h#emorrhag* or neoplasm* or disease* or myelopath* or myelitis or wound or degenerat* or tumo#r))

OR

TI (fractur* N3 (burst or chance or compression* or lumbar* or sacrum* or thoracic))

OR

AB (fractur* N3 (burst or chance or compression* or lumbar* or sacrum* or thoracic))

OR

TI (trauma* N3 myelopath*)

OR

AB (trauma* N3 myelopath*)

OR

TI (hangman* N2 fractur*)

OR

AB (hangman* N2 fractur*)

OR

(MH "Paraplegia")

(MH "Quadriplegia")

(MH "Hemiplegia")

OR

TI (paraplegi*) OR AB (paraplegi*)

OR

TI (quadriplegi*) OR AB (quadriplegi*)

OR

TI (tetraplegi*) OR AB (tetraplegi*)

OR

TI (hemiplegi*) OR AB (hemiplegi*)

OR

TI (SCI) OR AB (SCI)

OR

TI (paraparesis) OR AB (paraparesis)

OR

TI (paresis) OR AB (paresis)

OR

TI (paraly#ed) OR AB (paraly#ed)

OR

TI (quadripares*) OR AB (quadripares*)

OR

TI (syringomyelia) OR AB (syringomyelia)

AND

(MH "COVID-19+") OR (MH "COVID-19 Testing") OR (MH "COVID-19 Vaccines") OR (MH "Post-Acute COVID-19 Syndrome") OR (MH "COVID-19 Pandemic") OR (MH "SARS-CoV-2")

OR

((MH "Coronavirus") OR (MH "Coronavirus Infections")) AND (MH "Disease Outbreaks")

OR

TI (nCoV* or 2019nCoV or 19nCoV or COVID19* or COVID or SARS-COV-2 or SARSCOV-2 or SARS-COV2 or SARSCOV2 or "SARS coronavirus 2" or "Severe Acute Respiratory Syndrome Coronavirus 2" or "Severe Acute Respiratory Syndrome Corona Virus 2")

OR

AB (nCoV* or 2019nCoV or 19nCoV or COVID19* or COVID or SARS-COV-2 or SARSCOV-2 or SARS-COV2 or SARSCOV2 or "SARS coronavirus 2" or "Severe Acute Respiratory Syndrome Coronavirus 2" or "Severe Acute Respiratory Syndrome Corona Virus 2")

OR

TI ((new or novel or "19" or "2019" or Wuhan or Hubei or China or Chinese) N3 (coronavirus* or "corona virus*" or betacoronavirus* or CoV or HCoV))

OR

AB ((new or novel or "19" or "2019" or Wuhan or Hubei or China or Chinese) N3 (coronavirus* or "corona virus*" or betacoronavirus* or CoV or HCoV))

OR

TI (longCOVID* or postCOVID* or postcoronavirus* or postSARS*)

OR

AB (longCOVID* or postCOVID* or postcoronavirus* or postSARS*)

OR

TI ((coronavirus* or corona virus* or betacoronavirus*) N3 (pandemic* or epidemic* or outbreak* or crisis))

OR

AB ((coronavirus* or corona virus* or betacoronavirus*) N3 (pandemic* or epidemic* or outbreak* or crisis))

OR

TI ((Wuhan or Hubei) N5 pneumonia)

OR

AB ((Wuhan or Hubei) N5 pneumonia)
